# Plantar Pressure Distribution among Older Persons with Different Types of Foot and Its Correlation with Functional Reach Distance

**DOI:** 10.1155/2016/8564020

**Published:** 2016-11-17

**Authors:** Aisyah Mohd Said, Maria Justine, Haidzir Manaf

**Affiliations:** Department of Physiotherapy, Faculty of Health Sciences, Universiti Teknologi MARA, Puncak Alam Campus, 42300 Puncak Alam, Selangor, Malaysia

## Abstract

*Background.* Changes in biomechanical structures of human foot are common in the older person, which may lead to alteration of foot type and plantar pressure distribution. We aimed to examine how foot type affects the plantar pressure distribution and to determine the relationship between plantar pressure distribution and functional reach distance in older persons.* Methods.* Fifty community-dwelling older persons (age: 69.98 ± 5.84) were categorized into three groups based on the Foot Posture Index. The plantar pressure (max⁡*P*) and contact area were analyzed using Footscan® RSScan platform. The Kruskal-Wallis test was used to compare the plantar pressure between foot types and Spearman's correlation coefficient was used to correlate plantar pressure with the functional reach distance.* Results.* There were significant differences of max⁡*P* in the forefoot area across all foot types. The post hoc analysis found significantly lower max⁡*P* in the pronated foot compared to the supinated foot. A high linear rank correlation was found between functional reach distance and max⁡*P* of the rearfoot region of the supinated foot.* Conclusions.* These findings suggested that types of the foot affect the plantar maximal pressure in older persons with functional reach distance showing some associations.

## 1. Background

Aging process leads to changes in the biomechanical structure of the human foot. Several studies have shown the declination of muscle strength, range of motion, and ligament structural properties may cause abnormal foot posture in the older person [1–3]. In community-dwelling elderly, approximately 30% were reported as having foot problems [4], and these changes may later contribute to the development of conditions leading to foot pain [5–7] and impaired balance [1, 8] and gait performance [9, 10].

The foot posture can be categorized into three types which are normal, pronated, and supinated [11]. Normal foot type is a foot structure with an average arch and calcaneal angle inclination [12]. The pronated foot is characterized by a flat medial longitudinal arch and calcaneal eversion and varus [11, 13, 14]. Meanwhile, the supinated foot is classified as having a rigid high medial longitudinal arch with calcaneal inversion and rearfoot varus [13, 15, 16]. During walking, a neutral foot should have a mechanical advantage to adapt to ground surface while facilitating shock absorption and to function as a rigid fulcrum to push the body in space [17]. However, both abnormal foot postures (pronated and supinated) can adversely affect gait mechanics. The pronated foot is more loose-packed, causing the midtarsal joint to unlock during ambulation which allows the foot to act as a shock absorber [1, 10, 18] but may decrease the ability to act as a rigid lever. In contrast, the supinated foot is more rigid, which allows the foot to act as a more efficient fulcrum for forward motion but not as an efficient shock absorber [1, 18]. Therefore, older person with altered foot posture may have difficulty to perform proper gait mechanics and might lead to distribution changes of plantar pressure.

An individual with abnormal foot posture may have altered pressure distribution compared to the normal foot during static or dynamic pressure analysis. In older persons, the magnitude of forces and pressure is exerted by the heel, lateral forefoot, and hallux with greater relative duration of contact with the heel, midfoot, and metatarsophalangeal region [7]. Therefore, it is postulated that an older person with abnormal foot posture and balance performance may present with higher plantar pressure distribution than normal foot posture. However, to date, little attention has been paid to examining the effect of foot posture and plantar pressure distribution in older person. In addition, further investigation is required in order to assess other associated factors that may correlate well with plantar pressure distribution especially between abnormal foot postures.

Accordingly, this study has two specific aims: to examine how foot type (neutral, pronated, and supinated) affects the plantar pressure distribution among older person and to determine the relationship between plantar pressure distribution and functional reach distance in older persons. Thus, with different foot postures taken into account, it is hypothesized that those with pronated foot would give out the highest pressure during walking compared to other types of foot postures (neutral and supinated). We also hypothesized that the level of plantar pressure distribution demonstrates a correlation with the functional reach distance measured.

## 2. Methods

### 2.1. Participants

A power analysis (*α* = 0.05; *β* = 0.25) was conducted using the G-Power 3.0© program [19], where the power is set at 0.80 using *F* test ANOVA for repeated measures with in-between factor models. Therefore, a total of 50 participants (median age: 69 years, 25th to 75th percentile, and 65 to 73 years) were recruited from community areas that were mainly occupied with elderly. The inclusion criteria for participants were as follows: (1) no chronic orthopaedic conditions, for example, rheumatoid arthritis, severe knee osteoarthritis, or pain in the lower limb area, (2) no vestibular or neurological impairments, (3) no peripheral neuropathy or deficits due to diabetes or any systemic conditions, (4) ability to walk 10 meters continuously without aids, and (5) no involvement in any structured exercise classes of more than three times a week (physically inactive). The study protocol was approved by the institutional ethics committee of the university. All participants completed an informed consent form.

### 2.2. Foot Classification

A trained assessor screened the participants for types of the foot by using the six-item Foot Posture Index (FPI), a clinical diagnostic tool with a reliability coefficient of 0.61 for application on elderly [4] that can distinctively quantify the particular foot into neutral, pronated, or supinated posture [20]. The participant's foot was visually inspected during standing in a comfortable stance. From the assessments, we found that 19 participants (age: 65 years to 81 years) exhibited neutral foot, 15 (age: 60 years to 80 years) had a pronated foot, and 16 (age: 61 years to 85 years) had the supinated foot.

### 2.3. Testing Procedure

All testing procedures were conducted in a controlled environment that took place indoors and were done in the morning. First, the assessor recorded participant's body weight (kg), body height (m), body mass index (BMI) (kg/m^2^), and leg length (cm). The measurement of the lower limb length was done in supine lying position, and the marked points were from the anterior superior iliac spine (ASIS) to the distal part of the medial malleolus (true leg length). The lower extremities were positioned symmetrically before leg length measurement. Then, the assessor recorded participant's functional reach distance using the Functional Reach Test (FRT) [21]. FRT is chosen for balance assessment based on its psychometric characteristic to measure one's limit in anterior-posterior stability during reaching for maximum displacement while standing, which “integrates biomechanics, postural control, and proprioceptive feedback and correlates results of the higher risk of falling” [22]. The FRT has high reproducibility analysis of test-retest reliability with the intraclass correlation coefficient for interobserver of 0.98 [23]. The participant started by standing, but not touching a wall, with shoulder flexed 90°. The baseline measurements were taken at the third fingertips, and they were instructed to “reach as far as possible to the front without taking a step.” The scores were calculated by assessing the difference between the starting point and the end point of the reach and averaged by scores from three trials [21].

### 2.4. Dynamic Plantar Pressure Analysis

The dynamic plantar pressure distribution assessments were done by walking along a 3-meter track with a force plate placed in the middle of the track. Participants were instructed to walk barefooted on the plantar pressure plate platform (Footscan RSScan International) at their normal walking pace and cadence. The trial was repeated if the foot contact with the pressure platform was incomplete or if the participant targeted the platform. The platform was automatically calibrated as new data on weight and foot size were inserted before each test on each individual. This study focuses on max⁡*P* reading (maximum pressure beneath a specific area beneath the foot) and contact area (area corresponding to each evaluated area) expressed in N/cm^2^ for pressure and cm^2^ for area.

### 2.5. Data Management and Analysis

**Figure 1:**
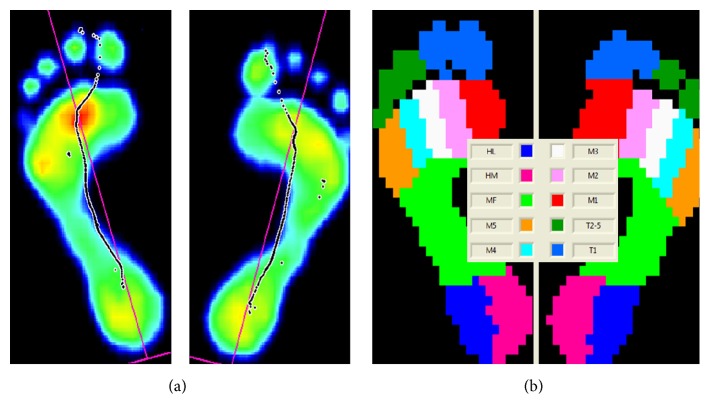
(a) Sample typical peak pressure image for a normal foot and (b) selection of anatomical zones in the Footscan software (HL: heel lateral; HM: heel medial; T1: big toe; T2–5: toes 2–5; M1, 2, 3, 4, and 5: metacarpals 1, 2, 3, 4, and 5).

The value recorded for each variable was the mean of the measurements corresponding to six steps with the dominant foot, recorded at the middle step in the series of steps. The foot dominance is determined through the question of which foot would be used to kick a ball rolled towards them, including demonstrations of it. Three successful walking trials for each subject were measured on a pressure plate with 4096 sensors with a scanning rate of up to 500 Hz. The maximal pressure and relative contact area data were extracted from the Footscan 7 USB2 Gait software (Figure 1). The forefoot area was calculated as the sum of the readings on toe 1, toes 2–5, and all 5-metatarsal areas (T1 + T2–5 + M1 + M2 + M3 + M4 + M5) and the rearfoot area was calculated as the sum of medial and lateral heel areas (HM + HL).

The nonparametric statistics were used to conduct the analysis using the SPSS statistical software (IBM Corp. Released 2011. IBM SPSS Statistics for Windows, Version 20.0. Armonk, NY: IBM Corp). Data were analyzed using nonparametric analysis based on our normality test using Shapiro-Wilk test where the data are abnormally distributed (*p* < 0.05) which is not sufficient to proceed using parametric analysis. The characteristics of participants were statistically described in median. Kruskal-Wallis test was used to compare whether the plantar pressure distribution and foot posture differed between the subject factors, with a post hoc test using Mann–Whitney test with Bonferroni correction done to analyze further the significant results. Spearman rank test was applied to correlate the plantar pressure variables functional reach distances. The level of significance of all statistical tests was set as *p* < 0.05.

## 3. Results

**Figure 2:**
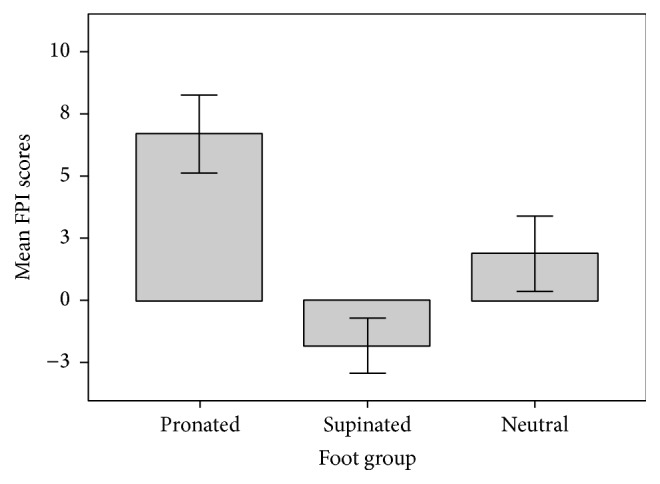
Error bars (+/− 1 SD) for FPI scores in different types of foot, showing no overlapping of mean scores within neutral, pronated, or supinated foot group. (FPI: Foot Posture Index.)

The characteristics of participants are presented in Table 1. There was no statistically significant difference found in age, body weight, body height, and other physiological characteristics between the three types of foot posture. The median BMI of the participants was 24.82 kg/m^2^ (25th to 75th percentile, 22.07 to 29.02) and functional reach distance was 22.67 (25th to 75th percentile, 16.63 to 27.79). Calculating the means and standard deviation for the FPI scores, no overlapping of standard deviation was reported within each group (Figure 2). The total FPI score ranges from 0 to 5 (mean = 1.89) in the neutral group, from 6 to 12 (mean = 6.73) in pronated group, and from −1 to −7 (mean = −1.81) in supinated group, where only one participant from pronated foot group was reported as having highly pronated foot.

### 3.1. Plantar Distributions between Types of Foot

Table 2 summarizes the participants' maximal pressure (max⁡*P*) and the contact areas of the forefoot, midfoot, and rearfoot across all three groups. Regarding the contact area, no significant difference (*p* < 0.05) was recorded across all types of foot. However, there were significant differences (*p* = 0.031) in max⁡*P* between foot types in the forefoot areas. The post hoc comparison revealed that the pronated foot has significant reduction of max⁡*P* in the forefoot area compared to the supinated foot group (*p* = 0.019), while the differences between the neutral foot and pronated foot and between neutral foot and the supinated foot were not significant (ALL, *p* > 0.05).

The max⁡*P* of the 4th metatarsal and 5th metatarsal subarea revealed significant differences between types of foot (*p* = 0.026 and *p* = 0.006, resp.). For the 4th metatarsal, post hoc comparison showed that the pronated foot led to a significant reduction of max⁡*P* compared to the neutral foot (*p* = 0.01). However, the differences between the supinated foot and neutral foot and between supinated and pronated foot were not significant (ALL, *p* > 0.05). For the 5th metatarsal, post hoc comparison revealed that pronated foot led to a significant reduction of max⁡*P* compared to supinated foot group (*p* = 0.002). In addition, the pronated foot resulted in a significant decrease of max⁡*P* compared to the neutral foot (*p* = 0.012). However, the difference between the supinated and neutral foot was not significant (*p* = 0.766).

### 3.2. Correlations between Plantar Pressure and Functional Reach Distance

The analysis was extended to a Spearman's rank correlation test between each type of foot and functional reach distance of the participants (Table 3). There is a high linear rank correlation with functional reach distance at the rearfoot area of the pronated foot (*r*_*s*_ = 0.604, *p* = 0.017). In the supinated foot, only the midfoot shows moderate linear rank correlation with functional reach distance (*r*_*s*_ = 0.504, *p* = 0.046) while others show no significant correlation.

## 4. Discussion

Our aim was to examine plantar pressure deviations in older persons with different types of the foot during dynamic analysis (walking). We present a few important findings. First, there was a significant decrease of max⁡*P* in the forefoot region of the pronated foot compared to the supinated foot, particularly in the 4th and 5th metatarsal. Second, the contact areas across all three types of foot show no significant differences as well as for total contact areas. Last, high to moderate linear rank correlation was found for functional reach distance with max⁡*P* at the rearfoot area of the pronated foot and between max⁡*P* and functional reach distance in the midfoot area of the supinated foot.

Our results demonstrated that max⁡*P* was significantly lower in a forefoot region of the pronated foot when compared to the supinated foot. In contrast, current finding is in line with a study [24] that reported a significant decrease in peak pressure and maximum force in their subjects with the low arch foot. However, there were differences in terms of age and footwear during testing in this study, which may have potentially influenced the loading on the medial portion of the foot.

Our result regarding the significantly lower max⁡*P* in only the pronated foot to supinated foot at the forefoot areas differs from our hypothesis where significantly lower pressure was predicted across all types of the foot, in which results of the neutral foot were taken as normal readings. Compared to previous studies [25–27], the plantar pressure distribution is significantly correlated with the types of foot or even any injury or changes that happened to the foot, and the plantar pressure distribution would also be affected [28, 29] especially during dynamic activities such as walking. Theoretically, older persons may display a flatter and pronated foot when compared to a younger adult [30] due to significant changes in the musculoskeletal and sensory characteristics of the foot throughout aging processes [31]. Thus, the peak pressure under the total foot would increase [32] during walking in order to compensate for the altered musculature of the foot. Therefore, the contradiction in our results may be explained by the mechanism of the gait that is confounded by the speed [33] as well as the pattern of making contact with the plantar areas during the barefoot walking [27, 34], which is hard to control due to the characteristics of the subjects, as being old. The age-dependent soft tissue properties of the plantar sole [32] may cause alteration in the walking pattern as the participants walk with their preferred pace and speed along the track. Thus, the speed and gait pattern might be limitations in this study that need further research to prove otherwise.

Another finding in this study is that the structure of the foot did not produce any alteration in the contact area under the plantar region across all foot types, while contradiction with previous study [35] reveals significant reduction of the weight-bearing area in supinated foot compared to normal. This is explained by associated deformities in supinated foot and a more rigid structure that limits the ability to absorb the impact compared to neutral foot [35].

Next, our findings indicate that plantar pressure is associated with functional reach distance in both pronated and supinated foot, but in different areas. The correlation was found between max⁡*P* at the rearfoot area of the pronated foot and that at the midfoot area in the supinated foot. We believe that this may be related to the joint compensation that occurs during the stance, which is most likely caused by the instability in the plantar area, leading to the shifting of the ground reaction force to concentrate towards more flexible area, in this case, rearfoot and midfoot area of pronated and supinated group, respectively. We hypothesized that the pronated foot would have better functional reach distance than the neutral and supinated foot due to a wider base of support, a typical presentation of a pronated foot [17, 36]. Even though the pronated foot was found to have better functional reach distance than those with the supinated foot [37], the plantar pressure result does not show any significant difference between these two-foot types. This is aligned with another study [13] that found no significant difference in functional reach distance between the pronated and neutral foot. But since the foot assessments, items analyzed, dynamic pressure, and footscan equipment were used and the participants' characteristics varied between studies, it is hard to extrapolate the data from one study to another. It would be interesting to know exactly the movement of the ground reaction force and the concentration of the balance point in this particular population during gait studies.

## 5. Limitations

This study has several limitations. First, the current findings obtained from a relatively small sample size limit the generalization of the findings to a larger population of older persons. Further studies should implement a larger population with a significant disability, including individuals at risk of falls. Second, gait strategies of the participants regarding speed, step length, or width were not controlled which might confound the result of the study. This may have affected the outcome of the current study.

## 6. Conclusions

With the use of plantar pressure analysis, participants showed statistically lower maximal pressure at the forefoot area of pronated foot compared to supinated foot. Accordingly, significantly lower maximal pressure was shown at the lateral sides of the pronated foot compared to supinated foot at 4th metatarsals and neutral foot at 5th metatarsals. We believe that more studies are needed to evaluate the variations of this pressure and their corresponding locations during other types of activity and to control the speed, cadence, and gait strategies to provide a better indication of the plantar pressure readings.

## Figures and Tables

**Table 1:** Characteristic of the participants (*N* = 50).

Demographic data	Median (25th–75th)			*p* value
	Neutral	Pronated	Supinated	
Age (years)	70.00 (67.00–73.00)	67.00 (63.00–71.00)	69.00 (63.00–73.75)	0.555
Height (m)	1.51 (1.47–1.54)	1.52 (1.48–1.55)	1.53 (1.49–1.58)	0.538
Weight (kg)	52.90 (43.40–67.20)	59.10 (53.80–73.00)	59.65 (52.80–73.00)	0.306
BMI (kg/m^2^)	24.56 (21.16–26.84)	27.53 (22.19–31.44)	25.61 (22.66–28.79)	0.314
Leg length (cm)	80.00 (75.00–83.50)	78.00 (77.00–83.00)	81.00 (76.25–83.75)	0.817
Functional reach distance (cm)	23.53 (16.00–29.00)	22.67 (15.53–28.67)	21.00 (17.13–26.38)	0.695

*p* value is significant at 0.05.

**Table 2:** Plantar pressure analysis among left and right foot between groups (neutral, pronated, and supinated foot); dynamic pressure (median (25th–75th)).

Parameters	Neutral	Pronated	Supinated	*p* value
Total contact area (cm^2^)	13.50 (13.10–13.90)	13.50 (13.10–14.30)	13.30 (12.80–14.30)	0.971
Contact area				
Forefoot	7.50 (7.50–8.30)	7.50 (7.20–8.30)	7.90 (7.50–8.30)	0.555
Midfoot	1.50 (1.10–1.50)	1.50 (1.50-1.50)	1.50 (0.88–1.50)	0.124
Rearfoot	3.00 (3.00-3.00)	3.00 (3.00-3.00)	1.50 (3.00-3.00)	0.189
max⁡*P* (N/cm^2^)				
Toe 1	2.20 (0–9.00)	0.90 (0.40–6.40)	1.40 (0–4.12)	0.875
Toe 2–5	2.00 (0.70–13.60)	2.20 (0.70–3.50)	2.75 (1.57–6.85)	0.465
Metatarsal 1	10.30 (5.30–31.20)	7.90 (4.40–18.90)	13.30 (8.85–34.65)	0.460
Metatarsal 2	16.10 (8.40–32.30)	7.70 (6.20–17.20)	12.75 (8.15–24.45)	0.177
Metatarsal 3	18.30 (13.20–27.30)	9.70 (4.20–13.20)	15.10 (7.95–35.08)	0.071
Metatarsal 4	12.50 (4.40–24.00)	4.20 (2.20–9.70)	11.90 (4.15–18.50)	0.026^*∗*^
Metatarsal 5	9.50 (2.20–18.50)	2.20 (1.10–4.20)	6.70 (3.70–13.10)	0.006^*∗∗*^
Medial heel	20.00 (14.30–43.80)	18.90 (9.20–22.90)	15.75 (12.05–31.67)	0.187
Lateral heel	13.90 (8.80–18.00)	10.30 (5.50–19.10)	13.05 (9.20–21.78)	0.387
Forefoot	91.80 (51.70–131.80)	48.40 (35.60–76.70)	76.25 (54.10–136.27)	0.031^*∗*^
Midfoot	5.90 (0.40–9.00)	3.50 (1.50–5.70)	4.60 (0.48–7.45)	0.971
Rearfoot	30.80 (25.70–71.90)	28.80 (14.70–38.70)	28.45 (22.27–44.58)	0.333

^*∗*^Significant at the 0.05 level (2-tailed).

^*∗∗*^Significant at the 0.01 level (2-tailed).

Analysis was done using Kruskal-Wallis test.

**Table 3:** Spearman Rank Correlation for max⁡*P* and anthropometrics and physiological factors in pronated, supinated, and neutral foot (*r*_*s*_ (*p* value)).

Type of foot	Subarea	Functional reach distance (cm)
Pronated	Forefoot	0.050 (0.859)
	Midfoot	0.107 (0.705)
	Rearfoot	**0**.**604** (**0**.**017**)^*∗*^
Supinated	Forefoot	0.102 (0.708)
	Midfoot	**0**.**504** (**0**.**046**)^*∗*^
	Rearfoot	0.332 (0.208)
Neutral	Forefoot	0.219 (0.369)
	Midfoot	−0.130 (0.597)
	Rearfoot	0.00 (1.00)

^*∗*^Significant at the 0.05 level (2-tailed).

## References

[1] Justine M., Ruzali D., Hazidin E., Said A., Bukry S. A., Manaf H. (2016). Range of motion, muscle length, and balance performance in older adults with normal, pronated, and supinated feet. *The Journal of Physical Therapy Science*.

[2] Deepashini H., Omar B., Paungmali A., Amaramalar N., Ohnmar H., Leonard J. (2014). An insight into the plantar pressure distribution of the foot in clinical practice: narrative review. *Polish Annals of Medicine*.

[3] Hemmati F., Forghany S., Nester C. (2014). The effects of pronated foot posture and medial heel and forefoot wedge orthoses on static balance in older people. *Journal of Foot and Ankle Research*.

[4] Menz H. B., Morris M. E., Lord S. R. (2006). Foot and ankle risk factors for falls in older people: a prospective study. *Journal of Gerontology*.

[5] Dufour A. B., Broe K. E., Nguyen U. (2009). Foot pain: is current or past shoewear a factor?. *Arthritis and Rheumatism*.

[6] Hagedorn T. J., Dufour A. B., Riskowski J. L. (2013). Foot disorders, foot posture, and foot function: the Framingham foot study. *PLoS ONE*.

[7] Janchai S., Chaiwanichsiri D., Silpipat N., Tiamprasitt J. (2008). Ageing feet and plantar arch characteristics of the Thai elderly. *Asian Biomedicine*.

[8] Menz H. B., Morris M. E., Lord S. R. (2005). Foot and ankle characteristics associated with impaired balance and functional ability in older people. *Journals of Gerontology: Medical Sciences*.

[9] Saleh A. G., Mohammed A. H. (2012). Plantar pressure distribution in patients with flexible flat foot, high arched foot and diabetic foot without neuropathy versus normal. *Bulletin of Faculty of Physical Therapy Cairo University*.

[10] Levinger P., Murley G. S., Barton C. J., Cotchett M. P., McSweeney S. R., Menz H. B. (2010). A comparison of foot kinematics in people with normal- and flat-arched feet using the Oxford Foot Model. *Gait & Posture*.

[11] Dahle L. K., Mueller M., Delitto A., Diamond J. E. (1991). Visual assessment of foot type and relationship of foot type to lower extremity injury. *Journal of Orthopaedic & Sports Physical Therapy*.

[12] Werd M. B., Knight E. L. (2010). *Athletic Footwear and Orthoses in Sports Medicine*.

[13] Cote K. P., Brunet M. E., Gansneder B. M., Shultz S. J. (2005). Effects of pronated and supinated foot postures on static and dynamic postural stability. *Journal of Athletic Training*.

[14] de Castro A. P., Rebelatto J. R., Aurichio T. R. (2011). The effect of gender on foot anthropometrics in older people. *Journal of Sport Rehabilitation*.

[15] Abbasi A., Tabrizi H. B., Sarvestani H. J., Bagheri K. (2011). Ground Reaction Forces attenuation in supinated and pronated foot during single leg drop-landing. *Annals of Biological Research*.

[16] Pezzan P. A. O., Sacco I. C. N., João S. M. A. (2009). Foot posture and classification of the plantar arch among adolescent wearers and non-wearers of high-heeled shoes. *Revista Brasileira de Fisioterapia*.

[17] Tiberio D. (1988). Pathomechanics of structural foot deformities. *Journal of American Physical Therapy Association*.

[18] Bonser R. J. (2012). *The effect of foot type on star-excursion and time-to-boundary measures during single-leg stance balance tasks [Ph.D. thesis]*.

[19] Faul F., Erdfelder E., Lang A.-G., Buchner A. (2007). G^∗^Power 3: a flexible statistical power analysis program for the social, behavioral, and biomedical sciences. *Behavior Research Methods*.

[20] Redmond A. C., Crane Y. Z., Menz H. B. (2008). Normative values for the Foot Posture Index. *Journal of Foot and Ankle Research*.

[21] Lin M.-R., Hwang H.-F., Hu M.-H., Wu H.-D. I., Wang Y.-W., Huang F.-C. (2004). Psychometric comparisons of the timed up and go, one-leg stand, functional reach, and Tinetti balance measures in community-dwelling older people. *Journal of the American Geriatrics Society*.

[22] Scena S., Steindler R., Ceci M., Zuccaro S. M., Carmeli E. (2016). Computerized functional reach test to measure balance stability in elderly patients with neurological disorders. *Journal of Clinical Medicine Research*.

[23] Duncan P. W., Weiner D. K., Chandler J., Studenski S. (1990). Functional reach: a new clinical measure of balance. *Journals of Gerontology*.

[24] Chuckpaiwong B., Nunley J. A., Mall N. A., Queen R. M. (2008). The effect of foot type on in-shoe plantar pressure during walking and running. *Gait and Posture*.

[25] Imaizumi K., Iwakami Y., Yamashita K. Analysis of foot pressure distribution data for the evaluation of foot arch type.

[26] Wenyan L., Goonetilleke R. S. Maximum plantar pressures, their locations and their use in footwear design.

[27] D’Août K., Pataky T., De Clercq D., Aerts P. (2009). The effects of habitual footwear use: foot shape and function in native barefoot walkers. *Footwear Science*.

[28] Neamţu M. C., Rusu L., Rusu P. F., Marin M., Neamţu O. M. (2012). Biomechanical disorders of foot in multiple sclerosis. *Romanian Journal of Morphology & Embryology*.

[29] Schepers T., Van der Stoep A., Van der Avert H., Van Lieshout E. M. M., Patka P. (2008). Plantar pressure analysis after percutaneous repair of displaced intra-articular calcaneal fractures. *Foot & Ankle International*.

[30] Chiu M.-C., Wu H.-C., Chang L.-Y., Wu M.-H. (2013). Center of pressure progression characteristics under the plantar region for elderly adults. *Gait & Posture*.

[31] Scott G., Menz H. B., Newcombe L. (2007). Age-related differences in foot structure and function. *Gait and Posture*.

[32] Bosch K., Nagel A., Weigend L., Rosenbaum D. (2009). From ‘first’ to ‘last’ steps in life—pressure patterns of three generations. *Clinical Biomechanics*.

[33] Chiu M.-C., Wu H.-C., Chang L.-Y. (2013). Gait speed and gender effects on center of pressure progression during normal walking. *Gait & Posture*.

[34] De Cock A., De Clercq D., Willems T., Witvrouw E. (2005). Temporal characteristics of foot roll-over during barefoot jogging: reference data for young adults. *Gait & Posture*.

[35] Fernández-Seguín L. M., Diaz Mancha J. A., Sánchez Rodríguez R., Escamilla Martínez E., Gómez Martín B., Ramos Ortega J. (2014). Comparison of plantar pressures and contact area between normal and cavus foot. *Gait and Posture*.

[36] Tsai L.-C., Yu B., Mercer V. S., Gross M. T. (2006). Comparison of different structural foot types for measures of standing postural control. *Journal of Orthopaedic and Sports Physical Therapy*.

[37] Hertel J., Gay M. R., Denegar C. R. (2002). Differences in postural control during single-leg stance among healthy individuals with different foot types. *Journal of Athletic Training*.

